# Zakażenie Wrodzone Wirusem Cytomegalii - Problem Wciąż Aktualny (na Podstawie Doświadczenia własnego Oraz Literatury)

**DOI:** 10.34763/devperiodmed.20182201.4957

**Published:** 2018-04-12

**Authors:** Dorota Lisowska-Mikołajków, Agata Mikołajków, Jędrzej Reczuch, Barbara Królak-Olejnik

**Affiliations:** 1 Katedra i Klinika Neonatologii, Uniwersytet Medyczny we Wrocławiu, Wrocławiu Polska; 2 Wydział Lekarski, Uniwersytet Medyczny im. Piastów Śląskich we Wrocławiu, Wrocławiu Polska

**Keywords:** wirus cytomegalii, ciężarna, płód, noworodek, diagnostyka, leczenie, profilaktyka, cytomegalovirus, pregnancy, fetus, newborn, diagnostics, treatment, prevention

## Abstract

Spośród czynników infekcyjnych wirus cytomegalii uważany jest za najczęstszą przyczynę postępującej utraty słuchu oraz zaburzeń neurologicznych u dzieci. Rosnąca liczba zakażeń wrodzonych spowodowanych tym wirusem wymusza podjęcie działań zmierzających do szybkiego ustalenia rozpoznania oraz wdrożenia leczenia. Wczesna diagnostyka oraz podjęte leczenie może skutkować, jeśli nie wyleczeniem, to zmniejszeniem następstw choroby. W działaniach diagnostyczno-leczniczych, aby zwiększyć ich skuteczność, muszą uczestniczyć nie tylko neonatolodzy ale również położnicy/perinatolodzy. W wielu krajach, w tym w Polsce, nie prowadzi się rutynowych badań w kierunku zakażenia wirusem cytomegalii u kobiet ciężarnych, co opóźnia diagnostykę, powoduje istotne wydłużenie czasu potrzebnego do włączenia leczenia przeciwwirusowego lub może je nawet uniemożliwić.

## Abstract (en)

As far as infectious factors are concerned, Cytomegalovirus is considered one of the most common causes of progressive hearing impairment and neurological disorders among children. The increasing number of CMV infections creates the necessity of quick diagnosis and treatment that may reduce the consequences or even completely resolve the condition. It is essential that the diagnostic team consists of not only neonatologists but also obstetricians/perinatologists. In many countries, including Poland, screening is not being carried out among pregnant women, which delays the diagnosis and the begining of antiviral treatment or might even indispose the therapy.

## Wprowadzenie

W ostatnich latach na świecie obserwuje się narastającą liczbę rozpoznawanych zakażeń wywołanych przez wirusa cytomegalii (ang. HCMV − humancytomegalovirus, CMV − cytomegalovirus). Częstość zakażeń tym wirusem jest zależna od statusu ekonomicznego danego państwa i jest tym większa im status ekonomiczny jest niższy. Jednocześnie też, w ostatnich latach, ludzki wirus cytomegalii stał się najczęstszym, spośród patogenów wirusowych, czynnikiem sprawczym zakażeń wrodzonych oraz jedną z głównych przyczyn zaburzeń neurologicznych, w tym upośledzenia umysłowego, a także utraty słuchu oraz zapalenia siatkówki i naczyniówki oka (chorioretinitis) u dzieci [1, 2, 3]. Zakażenie wrodzone wirusem CMV wiąże się aż z 30% umieralnością w tej grupie [2]. Szacuje się, że na całym świecie wrodzone zakażenie HCMV występuje z częstością 5-7/1000 żywych urodzeń. Około 10% zakażonych noworodków ma postać objawową, a pozostałych 90% ma postać bezobjawową zakażenia. W grupie noworodków z bezobjawowym zakażeniem, aż u 13,5% występują późne, groźne w skutkach następstwa. Najczęstszym z nich jest niedosłuch odbiorczy (neurosensoryczny) [1, 2, 3]. Ustalenie rozpoznania wrodzonego zakażenia HCMV konieczne jest w ciągu pierwszych trzech tygodni życia noworodka, co koreluje z czasem inkubacji wirusa (20-60 dni). Badania serologiczne oraz wirusologiczne wykonane w okresie późniejszym niż 20 dni po urodzeniu nie pozwalają już jednoznacznie na odróżnienie zakażenia wrodzonego HCMV od nabytego. W większości krajów, w tym w Polsce, nie prowadzi się rutynowych badań w kierunku zakażenia wirusem cytomegalii u kobiet ciężarnych. Podjęcie takiej decyzji przez Towarzystwa Ginekologiczne jest argumentowane: a) istnieniem udokumentowanej odporności w postaci obecności przeciwciał anty-CMV IgG (które jednak nie wyklucza możliwości zakażenia wrodzonego), b) nie każde zakażenie wewnątrzmaciczne prowadzi do wystąpienia zakażenia objawowego i późniejszych następstw u dziecka, c) nie ma ustalonego postępowania leczniczego w stosunku do płodu, d) badania rutynowe mogą wywoływać niepotrzebny niepokój u ciężarnej, e) zbyt wysokie koszty badań rutynowych w obliczu braku skutecznej interwencji terapeutycznej [3, 4].

Aktualizacja zagadnień związanych z zakażeniem cytomegalowirusem, jego replikacją, odpowiedzią immunologiczną, patogenezą, diagnostyką oraz możliwościami terapeutycznymi i pro+laktycznymi, może przyczynić się do zwery+kowania stanowiska dotyczącego działań pro+laktycznych, mających na celu ograniczenie liczby zakażeń wrodzonych HCMV. Wydaje się, że znalezienie skutecznych metod pro+laktyki, rozpoznawania oraz leczenia wrodzonych zakażeń wirusem cytomegalii jest jednym z najpoważniejszych wyzwań współczesnej medycyny. Dlatego też, opracowanie skutecznej szczepionki, stanowi jeden z priorytetów Światowej Organizacji Zdrowia. Jednak do czasu opracowania takiej szczepionki jedyną formą zapobiegania transmisji HCMV, szczególnie wśród kobiet w okresie prokreacyjnym, jest edukacja [5, 6].

**Cytomegalia** jest ostrą chorobą wirusową, wywołaną przez ludzki wirus cytomegalii, należący do grupy DNA wirusów z rodziny Betaherpes. Ze wszystkich opisanych do tej pory herpeswirusów HCMV jest prawdopodobnie przyczyną największej liczby zachorowań i śmiertelności. Ocenia się, że przeciwciała świadczące o przebytym zakażeniu tym wirusem posiada 40-80% ludzi na świecie, i to zarówno w środowiskach o wysokim, jak i o niskim standardzie socjoekonomicznym. Mimo szerokiego rozpowszechnienia wirusa cytomegalii w świecie, częstość występowania przeciwciał anty-CMV w populacjach różnych kontynentów nie jest jednakowa: w Europie wykrywa się je u 40% populacji, w USA –u 70%, a w krajach Trzeciego Świata u nawet u 100% badanych (6). W Polsce, u kobiet w wieku rozrodczym, w okresie prekoncepcyjnym przeciwciała anty-CMV wykryto u blisko 80% badanych, a ich odsetek był tym większy im wyższy był wiek badanych kobiet (74,3-94,3%) (4).

Zarówno u dzieci, jak i u dorosłych większość infekcji HCMV oraz ich reaktywacji ma przebieg bezobjawowy [6, 7, 8]. Zakażenia bezobjawowe lub utajone notowane są u 85-90% badanych dzieci, a manifestujące się objawami chorobowymi u 10-15%. U 10-20% badanych dzieci z wrodzonym zakażeniem wirusem cytomegalii objawy ze strony ośrodkowego układu nerwowego (OUN) lub narządu wzroku i słuchu mogą rozwinąć się w późniejszym okresie życia. U osób z wrodzonymi lub nabytymi zaburzeniami odpowiedzi immunologicznej (zakażonych ludzkim wirusem niedoboru odporności − *ang. human immunodeficiency virus* − HIV, po leczeniu immunosupresyjnym), a także u noworodków oraz płodów, istnieje duże ryzyko wystąpienia ciężkiej postaci choroby, co może prowadzić do patologii wielonarządowej, najczęściej obejmującej ośrodkowy układ nerwowy, w tym narząd wzroku i słuchu [4, 6, 9].

Wirus cytomegalii ma długi cykl replikacyjny, a zakażone nim komórki ulegają znacznemu powiększeniu, czemu towarzyszy pojawienie się jądrowych i cytoplazmatycznych ciałek wtrętowych. HCMV, podobnie jak inne wirusy z rodziny Herpesviridae, charakteryzuje się zdolnością do latencji w makrofagach, w subpopulacji CD8 limfocytów T oraz w komórkach gruczołów wydzielania wewnętrznego, jak również możliwością okresowej reaktywacji [9]. Wirus CMV może się rozprzestrzeniać w płynach ustrojowych (mocz, łzy, sperma, ślina, krew, mleko kobiece, wydzielina szyjki macicy). Z tego też powodu zarówno ostre pierwotne zakażenie ciężarnej, ale również zakażenie wtórne jako konsekwencja „obudzenia” i reaktywacji wirusa latentnego lub też reinfekcji (zakażenia wirusem cytomegalii o innym serotypie) stanowią istotne ryzyko zakażenia płodu i związane z nim konsekwencje w postaci jego obumarcia, poronienia, porodu przedwczesnego lub ciężkich zaburzeń neurologicznych w przyszłości.

W wielu zachorowaniach, początkowo bezobjawowych, istnieje ryzyko ujawnienia się wrodzonego zakażenia dopiero po latach. Przez wiele miesięcy lub lat bezobjawowy „nosiciel wirusa” (którym są najczęściej dzieci w wieku żłobkowym i przedszkolnym) wydalając go, stanowi istotne zagrożenie dla otoczenia. Za grupę szczególnie narażoną uważa się opiekunki dziecięce w żłobkach i przedszkolach, personel medyczny w oddziałach pediatrycznych i noworodkowych oraz personel laboratoriów analitycznych.

Wczesne rozpoznanie wrodzonego zakażenia CMV (zarówno objawowego jak i bezobjawowego) jest możliwe w przypadku wdrożenia rutynowej diagnostyki w kierunku zakażenia tym wirusem zarówno u ciężarnej jak i u noworodka.

**Wrodzone zakażenie CMV** jest jedną z najczęstszych wirusowych infekcji wewnątrzmacicznych (10-40%) [8]. Postać objawowa zakażenia występuje, w zależności od cytowanych źródeł, od 5-10%, a nawet 12,7% [1, 2, 3, 4, 5] dzieci z tą chorobą i najczęściej charakteryzuje się następującymi objawami klinicznymi: małogłowiem, wybroczynami, wysypką typu „blueberry mu; n”, hepatosplenomegalią, zaburzeniami dystrybucji napięcia mięśniowego (wzmożone lub obniżone), drgawkami. Często stwierdza się niedosłuch czuciowo-odbiorczy, zaburzenia oczne (nieprawidłowości tylnego odcinka gałki ocznej: retinochorioiditis, blizny w plamce żółtej i/lub siatkówce, zanik nerwu wzrokowego, zapalenie błony naczyniowej, zaćma, blizny rogówki). Uważa się, że z wrodzonym zakażeniem CMV mogą współwystępować: zez, małoocze, brak gałki ocznej [1, 5, 6]. Odchylenia w wynikach badań laboratoryjnych obejmują: niedokrwistość, małopłytkowość, neutropenię, hyperbilirubunemię, cholestazę wewnątrzwątrobową, wzrost aktywności aminotransferaz, podwyższone stężenie białka w płynie mózgowo-rdzeniowym (PMR). W wynikach badań obrazowych ośrodkowego układu nerwowego często stwierdza się zwapnienia w istocie białej okołokomorowej lub podwyściółkowo usytuowane zmiany torbielowate, waskulopatię wzgórzowo-prążkowiową, poszerzenie układu komorowego nadnamiotowego. Z zakażeniem wrodzonym CMV mogą wiązać się wady rozwojowe mózgowia w postaci: lissencefalii, pachygyrii, polimykrogyrii, hipoplazji mózgu przebiegającej z lub bez hipoplazji móżdżku [10, 11, 12, 13].

Objawowa cytomegalia wrodzona wiąże się z 90% ryzykiem następstw neurologicznych, co może skutkować w przyszłości niedorozwojem umysłowym, ciężkim, postępującym zaburzeniem słuchu o charakterze czuciowo-odbiorczym, ciężkimi powikłaniami ocznymi. U dzieci z utajonym zakażeniem zagrożenie upośledzeniem słuchu wynosi ok. 10-15%. U 10% z nich może rozwinąć się później, aż do 5-7 roku życia. Zmysł słuchu jest jednym z najistotniejszych zmysłów w rozwoju dziecka i to zarówno w aspekcie rozwoju mowy, rozwoju umysłowego, jak i poznawczego. Wczesne rozpoznanie choroby jest istotne tym bardziej, że niedosłuch odbiorczy, będący skutkiem wrodzonego zakażenia CMV może mieć charakter postępujący.

Niecharakterystycznymi, ale występującymi w przebiegu zakażenia wrodzonego CMV są: wcześniactwo, wewnątrzmaciczne zahamowanie wzrostu płodu (IUGR/ FGR − i*ntrauterine vel fetal growth restriction*), zmiany charakterystyczne dla retinopatii wcześniaczej, odwarstwienie siatkówki w przebiegu retinitis, zespół mononukleozopodobny, opóźnienie rozwoju psychoruchowego, małogłowie wtórne, nieprawidłowości w zakresie przewodu pokarmowego oraz układu krążenia [2, 5, 6].

Ze względu na brak dostatecznych danych epidemiologicznych dotyczących zakażenia wrodzonego HCMV wydaje się zasadnym, by diagnostykę w tym kierunku poszerzyć u noworodków z następującymi objawami:

− wcześniactwo,
− hypotro+a,
− nasilona, przedłużająca się żółtaczka noworodka,
− objawy oczne (retinochorioiditis, blizny w plamce żółtej i/lub siatkówce, zanik nerwu wzrokowego, zapalenie błony naczyniowej, zaćma, blizny rogówki),
− nieprawidłowy wynik przesiewowego badania słuchu,
− zmiany w mózgowiu (ventriculomegalia, wodogłowie, zwapnienia, wady migracji neuroblastów, małogłowie),
− uogólnione zakażenie z towarzyszącą organomegalią,
− zmiany skórne (wybroczyny , wysypka typu „ blueberry muffin”).

## Diagnostyka

Dziecko: „Złotym standardem” w diagnostyce zakażenia wrodzonego HCMV jest badanie z wykorzystaniem metody PCR. Badania serologiczne z oceną swoistych przeciwciał są trudne do interpretacji. Nie zawsze bowiem brak swoistych przeciwciał klasy IgM wyklucza zakażenie wrodzone, zwłaszcza, jeśli do zakażenia doszło w pierwszym lub drugim trymestrze ciąży lub w wyniku reaktywacji wirusa latentnego.

Podstawą rozpoznania wrodzonego zakażenia CMV jest stwierdzenie obecności wirusa w moczu lub ślinie w pierwszych 20-21 dniach życia noworodka, co wynika z okresu jego inkubacji. Stwierdzenie obecności wirusa CMV w materiale biologicznym po 21 dobie życia noworodka nie daje zatem możliwości odróżnienia zakażenia wrodzonego od nabytego. Czułość tej metody jest bardzo wysoka również u noworodków przedwcześnie urodzonych [5, 6]. Z uwagi na wysokie stężenie wirusa w moczu, łatwą dostępność próbki moczu lub śliny oraz wysoką czułość testu, badanie metodą PCR jest uznane za „złoty standard”. Ujemny wynik badania pozwala bowiem na wykluczenie wrodzonego zakażenia CMV. Badanie krwi ilościową metodą PCR ma zdecydowanie mniejsza czułość, jednak u pacjentów z objawową postacią choroby jest cennym badaniem uzupełniającym. Pozwala bowiem na ocenę skuteczności leczenia przeciwwirusowego, natomiast u pacjentów z zakażeniem bezobjawowym CMV może mieć znaczenie prognostyczne. Uważa się, że ryzyko wystąpienia w późniejszym okresie życia objawów neurologicznych wzrasta o 50%, jeśli liczba kopii wirusa we krwi wynosi powyżej 1,2 x10/4 kopii/ml i odpowiednio ryzyko uszkodzeń słuchu wzrasta o 50%, jeśli liczba kopii wirusa we krwi wynosi powyżej 1,7 x10/4 kopii/ ml [5, 15]. Podobnie dodatni wynik badania metodą PCR w PMR przemawia za neuroinfekcją i koreluje z niekorzystnym rokowaniem [5, 16]. Jeśli liczba kopii wirusa w moczu nie przekracza 500 (0,5x10/3) kopii/ ml, nie należy spodziewać się jego obecności w większej liczbie w pozostałych płynach ustrojowych. Jeśli liczba kopii wirusa oznaczana metodą PCR w moczu wynosi powyżej 500 (0,5x10/3) kopii/ml, wówczas wskazane jest poszerzenie diagnostyki o badanie innych płynów ustrojowych jak krew czy płyn mózgowo-rdzeniowy.

Stosowane powszechnie badania serologiczne z oceną IgG i IgM CMV, są jedynie pośrednimi metodami diagnostyki wrodzonego zakażenia CMV. Mają wartość diagnostyczną jedynie w przypadku, gdy:

- stwierdzamy brak swoistych przeciwciał w klasie IgG i IgM zarówno w surowicy matki jak i dziecka, co pozwala wykluczyć zakażenie, lub
- w przypadku, gdy u noworodka do 21 doby życia stwierdzamy obecność swoistych IgM, co jest dowodem na zakażenie wrodzone.

Obecność swoistych przeciwciał antyCMV w klasie IgM jest stwierdzana jedynie u 20-70% zakażonych dzieci [5, 16]. Pozostałe warianty odpowiedzi serologicznej wymagają wery+kacji. Brak przeciwciał w klasie IgM, przy obecności IgG nie wyklucza zakażenia wrodzonego CMV [5]. Zatem powtarzanie badań serologicznych u dziecka bez równoczesnych badań molekularnych (PCR w moczu) na początku diagnostyki jest błędem w postępowaniu. Powoduje bowiem opóźnienie rozpoznania lub nawet je uniemożliwia, co może skutkować opóźnieniem lub odstąpieniem od leczenia.

W przypadku rozpoznania wrodzonego zakażenia CMV konieczne jest rozszerzenie diagnostyki zarówno laboratoryjnej jak i obrazowej w celu oceny zaawansowania choroby i ustalenia wskazań do leczenia przeciwwirusowego. Wśród badań laboratoryjnych wskazane jest wykonanie: morfologii krwi z rozmazem, aktywności transaminaz, stężenia bilirubiny z rozdziałem, zaś u dzieci z objawami neurologicznymi badanie ogólne płynu mózgowo-rdzeniowego. Należy także rozważyć wykonanie badań molekularnych (PCR) z krwi i płynu mózgowo-rdzeniowego. Wśród badań obrazowych OUN należy wykonać badanie ultrasonograficzne mózgowia (usg) i/lub rezonans magnetyczny mózgowia (MRI). Badanie dna oka oraz pełne badanie audiologiczne w postaci wywołanych potencjałów słuchowych pnia mózgu pomoże wykluczyć zmiany dotyczące narządu wzroku i słuchu.

Poszerzenie diagnostyki obrazowej o badanie MRI mózgu jest wskazane nie tylko w przypadku zmian w badaniu usg przezciemiączkowym, ale powinno być wykonywane w każdym zakażeniu objawowym, w celu szczegółowej oceny mózgowia, w tym diagnostyki wad kory mózgowej. Do najczęstszych nieprawidłowości w obrazie mózgu u noworodków z wrodzonym zakażeniem HCMV należą: zwapnienia w istocie białej okołokomorowej, podwyściółkowo usytuowane zmiany torbielowate, waskulopatia wzgórzowo-prążkowiowa, poszerzenie układu komorowego nadnamiotowego, wady rozwojowe mózgowia w postaci lissencefalii, pachygyrii, polimykrogyrii, hipoplazji mózgu przebiegającej z lub bez hipoplazji móżdżku [16, 17, 18].

Matka: W związku z faktem, że największe ryzyko wystąpienia ciężkich powikłań zakażenia CMV u płodu jest związane z zakażeniem pierwotnym u matki, głównym celem diagnostyki wrodzonych zakażeń CMV jest potwierdzenie lub wykluczenie tego zakażenia u ciężarnej. Najpewniejszą metodą potwierdzenia jest stwierdzenie serokonwersji, czyli wykrycie swoistych przeciwciał u uprzednio seronegatywnej ciężarnej. Obecność swoistych przeciwciał w klasie IgM potwierdza aktualne lub niedawno przebyte zakażenie. Przeciwciała tej klasy utrzymują się zwykle 3-6 miesięcy, a czasem powyżej 12 miesięcy od momentu zakażenia. W takim przypadku pomocnym jest oznaczenie awidności przeciwciał IgG. Przeciwciała IgG produkowane w wyniku pierwotnego zakażenia mają niską siłę wiązania antygenu (niską awidność). W miarę rozwoju zakażenia, po około 20 tygodniach, powyżej 60% przeciwciał wykazuje już wysoką awidność [15]. Zatem metoda ta służy do różnicowania zakażenia pierwotnego i wtórnego oraz pomocna jest w sytuacjach długotrwałego utrzymywania się swoistych IgM lub wyników fałszywie dodatnich. Należy jednocześnie wspomnieć o możliwym fenomenie utrzymywania się niskiej awidności powyżej 18 tygodni, co przy przetrwaniu IgM lub fałszywie dodatnim wyniku IgM, może skutkować błędnym rozpoznaniem zakażenia pierwotnego [19].

W sytuacji potwierdzenia zakażenia pierwotnego u matki na podstawie obecności anty CMV IgM i/lub anty CMV IgG o niskiej awidności istnieje ryzyko zakażenia płodu. W takiej sytuacji badaniem z wyboru jest oznaczenie obecności wirusa w płynie owodniowym (AF-*amniotic fluid*). AF do badań wirusologicznych w kierunku CMV winien być pobrany najlepiej pomiędzy 21-22 Hbd, ale co najmniej 6 tygodni po zakażeniu matki [20]. Powyższe wynika z faktu, że wirus jest wydalany drogą nerek, a nerki u płodu zaczynają efektywnie produkować mocz po 21 Hbd. Ponadto wirus CMV jest wydalany z moczem płodu na wykrywalnym poziomie po upływie 6-9 tygodni od zakażenia ciężarnej [15, 21]. Można dokonać próby izolacji wirusa w hodowlach komórkowych, jednak złotym standardem jest oznaczenie obecności materiału genetycznego CMV metodą PCR. W badaniach Lazzarotto [20, 21] wykazano, że obecność kopii wirusa CMV w AF na poziomie powyżej 10/3 kopii/ml w 100% wskazuje na istnienie zakażenia wrodzonego, a wartość powyżej 10/5 kopii/ml wiąże się z wysokim ryzykiem urodzenia dziecka z objawami zakażenia, zaś liczba kopii wirusa poniżej 500/ml z dużym prawdopodobieństwem wyklucza wystąpienie objawowej cytomegalii u noworodka. Pomocną metodą jest rutynowa ultrasonogra+a płodu. Według zaleceń Polskiego Towarzystwa Ginekologicznego (PTG) powinno być ono wykonywane 3-krotnie: w 12, 20 i 30 Hbd. Można wówczas wykazać szereg zmian mogących sugerować zakażenie CMV w postaci: hypotro+i płodu, małogłowia, poszerzenia komór mózgu, zwapnień okołokomorowych, zwiększonej echogeniczności jelit, wielowodzia, obrzęku płodu, wysięku opłucnowego, pogrubienia łożyska [4, 5, 9]. Stwierdzenie powyższych zmian w obrazie usg powinno być wskazaniem do badań mających na celu potwierdzenie lub wykluczenie zakażenia CMV. Czułość metody usg wynosi jednak ok. 8,5-14,9% [22, 23, 24]. Wśród czynników prognostycznych objawowej cytomegalii wrodzonej wymienia się także wynik ilościowego PCR we krwi płodu, ponadto liczbę płytek krwi oraz stężenie IgM we krwi płodu. Jednak konieczne są dalsze badania nad kliniczną przydatnością ww. metod.

Wydaje się zatem, że najkorzystniejsze jest oznaczenie stężenia przeciwciał IgG i IgM CMV w okresie prekoncepcyjnym, następnie przynajmniej 2. razy w czasie trwania ciąży (I i III trymestr). W razie potrzeby oznaczamy także awidność IgG.

**Co powinno zaniepokoić położnika ?**

− serokonwersja odczynów serologicznych z ujemnych na dodatnie, wskazująca na zakażenie pierwotne,

− wzrost stężenia przeciwciał IgG CMV, zwłaszcza przy niskiej awidności, wskazujący na reaktywację lub reinfekcję zakażenia. (Rycina 1).

Rycina 1. przedstawia schemat diagnostyki zakażenia wirusem cytomegalii u ciężarnej oraz jej płodu opracowany na podstawie schematów Lazzarotto i Munro [21, 37]. Z uwagi na zwięzłą formę schematu, która wymusza uogólnienia i nie uwzględnia wszystkich możliwych wariantów, należy dodać, że izolowana obecność IgG (IgG dodatnie i IgM ujemne), nie wyklucza zakażenia wrodzonego, ze względu na możliwą reinfekcję innym serotypem wirusa, lub reaktywację wirusa uśpionego. Ponadto ujemny wynik badania metodą PCR z płynu owodniowego nie wyklucza zakażenia płodu, do którego może dojść po wykonaniu amniopunkcji.

**Ryc. 1:**
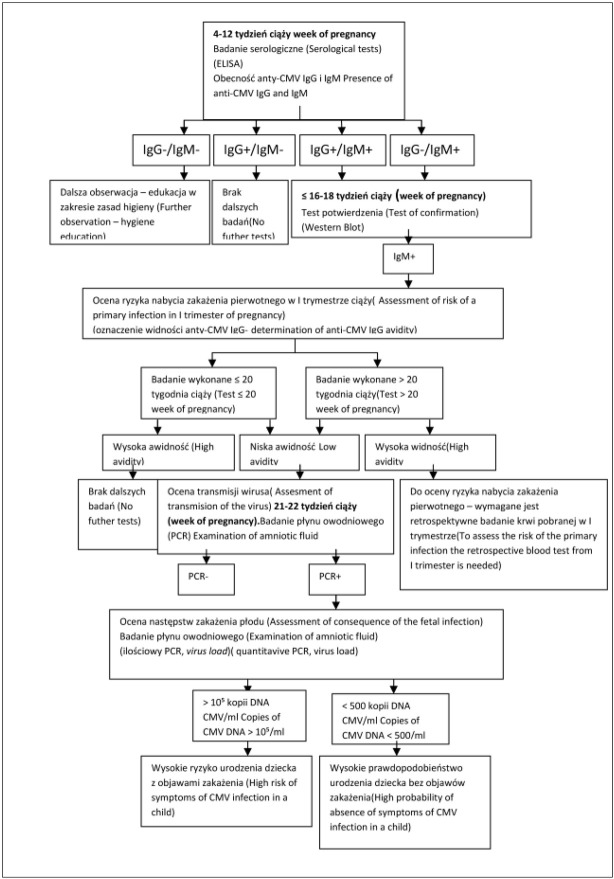
Schemat diagnostyki laboratoryjnej zakażenia CMV w ciąży, na podstawie Lazzarotto i Munro [21, 37]. Fig. 1. Laboratory examination of CMV infection Turing pregnancy, based on Lazzarotto and Munro [21, 37].

## Leczenie i profilaktyka

### Szczepienie

Duże nadzieje pokładane sąw możliwości kontroli zakażenia CMV na drodze szczepień. Oczekuje się, że szczepienie powinno chronić przed transmisją wirusa u kobiet w ciąży (zakłada się immunizację kobiet w wieku rozrodczym oraz 12-letnich dziewcząt) oraz zmniejszyć transmisję wśród dzieci w żłobkach i przedszkolach, a także transmisję dziecko-dorosły (zakłada się szczepienie powszechne niemowląt i dzieci) [25]. Idealna szczepionka powinna stymulować zarówno odpowiedź humoralną jak i komórkową. Jednak pomimo intensywnych badań prowadzonych od lat 60-tych ubiegłego wieku, nie udało się opracować skutecznej i bezpiecznej szczepionki przeciwko wirusowi cytomegalii. Badania nadal trwają.

### Bierna immunizacja

W profilaktyce zakażeń wrodzonych CMV stosowana jest bierna immunizacja. Mechanizm działania HIG (*HyperImmune Globulin*) obejmuje neutralizację cząsteczek wirusa, podwyższenie aktywności komórek NK, cytotoksyczności, blokowanie procesu przyłączania wirusa. W badaniach Nigro [26] wykazano, że podawanie immunoglobuliny kobietom, u których zdiagnozowano pierwotne zakażenie CMV zmniejszyło ryzyko infekcji u płodu. Podawanie HIG wywiera korzystny wpływ w sposób bezpośredni, poprzez przedostanie się przeciwciał do krążenia płodu oraz pośrednio poprzez redukcję pogrubienia łożyska, co poprawia jego funkcje odżywcze w stosunku do płodu [27, 28]. Pomimo, że w kilku doniesieniach opisywano korzyści z podaży HIG, to brak jest nadal ustaleń odnośnie dawki i schematu podaży. W badaniu z randomizacją (Revello 2014) nie wykazano znaczącej korzyści z podaży HIG, obserwowano natomiast powikłania położnicze. Nadal trwają próby kliniczne, które miejmy nadzieję określą rolę HIG w zapobieganiu infekcji wrodzonej. Aktualnie nie rekomenduje się rutynowego stosowania HIG u ciężarnych, podobnie jak stosowania rutynowej terapii przeciwwirusowej u ciężarnych [6].

### Leki

W chwili obecnej nie ma skutecznego leku, który pozwoliłby na wyeliminowanie wirusa z organizmu osoby zakażonej. Stosowane leki jedynie hamują namnażanie wirusa we krwi, a po ich odstawieniu często obserwuje się wystąpienie lub stopniowe narastanie wiremii. Wyniki badań z randomizacją [29, 30] wykazały, że zastosowanie 6-tygodniowego cyklu leczenia ganciclowirem, w stosunku do placebo, zmniejsza odsetek uszkodzeń słuchu oraz poprawia rozwój psychoruchowy u dzieci z objawową cytomegalią wrodzoną, z zajęciem OUN. W czasie leczenia obserwuje się ustąpienie wiremii, wirurii oraz objawów uszkodzenia narządowego, w tym ustępowanie zmian skórnych, hepatosplenomegalii, cofanie się odchyleń w badaniach laboratoryjnych w postaci neutropenii, trombocytopenii, ustępowanie zmian na dnie oczu oraz zmniejszanie niedosłuchu [31, 32]. Badania wykazały również, że wydłużenie terapii z 6 tygodni do 6, a nawet 12 miesięcy przyniosło lepsze efekty odległe w postaci zmniejszenia odsetka niedosłuchu oraz dało lepsze wyniki w ocenie neurorozwojowej [33]. Zatem leczenie przeciwwirusowe powinno być rozpoczęte jak najwcześniej po urodzeniu (należy je rozpocząć w pierwszych 28-30 dniach życia) i trwać minimum 6 tygodni, a biorąc pod uwagę długofalowe korzyści z terapii dłuższej, kilkumiesięcznej, należy rozważyć przedłużenie leczenia, zwłaszcza przy jego dobrej tolerancji, do 3-6 miesięcy [5, 6]. Rekomendacje międzynarodowe sugerują wyłączne leczenie doustne valganciclovirem (VGCV), przez okres do 6 miesięcy [6].

Wskazania do leczenia przeciwwirusowego u noworodków według Marshall i Stamminger to [22]:

1. Pełnoobjawowa cytomegalia wrodzona (postać uogólniona).
2. Cytomegalia wrodzona z zajęciem OUN (małogłowie, zwapnienia, poszerzenie układu komorowego, wasculopatia, torbiele podwyściółkowe, drgawki).
3. Cytomegalia wrodzona ze zmianami zapalnymi na dnie oczu.
4. Cytomegalia wrodzona z izolowanym niedosłuchem.

Do leczenia przeciwwirusowego kwalifikowane są noworodki z wrodzonym objawowym zakażeniem CMV. Brak jest wskazań do leczenia u noworodków z zakażeniem skąpoobjawowym. Leczenie przeciwwirusowe dzieci z izolowanym niedosłuchem, bez innych objawów klinicznych infekcji wrodzonej jest dyskusyjne i nie jest rekomendowane przez międzynarodowy panel ekspertów rutynowo, z uwagi na niewystarczające dowody skuteczności (Rawlinson 2017), choć polskie rekomendacje są w tym względzie odmienne (Pokorska-Śpiewak 2016).

Obecnie zarejestrowane są cztery leki przeciwwirusowe przeznaczone do leczenia zakażeń wywołanych przez CMV: ganciclovir (GCV), valganciclovir (VGCV), cidofovir (CDV), i foscarnet (PFA). Lekiem z wyboru jest ganciclovir i valganciclovir, a foscarnet najczęściej stosuje się w przypadku zakażeń opornych na GCV. W leczeniu stosuje się dożylnie ganciclovir (5 mg/kg/dawkę co 12 godzin) lub valganciklovir doustnie (16 mg/kg/dawkę co 12 godzin). Oba preparaty mają porównywalną skuteczność, oba przenikają do płynu mózgowo-rdzeniowego [5, 6, 33, 34]. Stosowanie gancicloviru wiąże się z potrzebą dostępu naczyniowego oraz koniecznością hospitalizacji, natomiast leczenie z zastosowaniem valgancicloviru można prowadzić w warunkach ambulatoryjnych, co umożliwia istotne obniżenie kosztów terapii oraz zmniejszenie jego uciążliwości. W trakcie leczenia obu preparatami należy monitorować stężenie leku we krwi, kontrolować systematycznie (raz w tygodniu) morfologię z rozmazem białokrwinkowym, próby czynnościowe wątroby i nerek. U noworodków wcześniaczych częściej zachodzi potrzeba wydłużenia odstępu miedzy dawkami leku do 18 godzin. W przypadku wystąpienia objawów niepożądanych leczenia w postaci neutropenii poniżej 500 (neutrofile <0,5x10/9/l), przerywamy leczenie do czasu wzrostu liczby granulocytów powyżej 800 (>0,8x10/9/l); w przypadku trombocytopenii poniżej 50 000 (<50x10/9/l) przerywamy leczenie do czasu wzrostu liczby płytek krwi powyżej 75 000 (>75x10/9/l) [5, 6, 22].

Główną drogą eliminacji gancicloviru jest jego wydalanie w formie niezmienionej przez nerki. Dlatego w przypadku pacjentów z zaburzeniami czynności nerek, którzy sąw grupie podwyższonego ryzyka toksyczności leku (szczególnie toksyczności hematologicznej) dawkę gancicloviru należy zmniejszyć. Stwierdzono bowiem, że całkowity klirens gancicloviru w ustroju jest skorelowany w sposób liniowy z klirensem kreatyniny, a ten z kolei koreluje z poziomem kreatyniny w surowicy. U pacjentów z zaburzeniami czynności nerek okres półtrwania leku w fazie eliminacji może ulec wydłużeniu nawet kilkukrotnie. Zaburzenia czynności wątroby nie mają znaczącego wpływu na farmakokinetykę gancicloviru i dlatego nie ma specyficznych zaleceń dotyczących zmiany dawkowania leku [35]. W Klinice Neonatologii we Wrocławiu opracowano schemat postępowania leczniczego. Z uwagi na stosowanie w leczeniu wrodzonego zakażenia HCMV preparatów leczniczych (ganciclovir, valganciclovir) w sposób odmienny niż opis w charakterystyce produktu leczniczego („o+ label use”), przed rozpoczęciem leczenia jesteśmy zobowiązani uzyskać od rodziców dziecka stosowną zgodę na proponowane leczenie.

Jeśli u noworodka HCMV znajduje się w moczu w liczbie powyżej 500 kopii wirusa/ml poszerzamy diagnostykę laboratoryjną metodą PCR o inne płyny ustrojowe. W zależności od uzyskanych wyników kontrolujemy wskaźniki laboratoryjne oraz wykonujemy badania obrazowe i narządów zmysłów (okulistyczne, audiologiczne) zgodnie z rekomendacjami przytoczonymi powyżej. W przypadku cytomegalii pełnoobjawowej (postaci uogólnionej) lub z zajęciem OUN (w tym zajęcie narządów zmysłów), w leczeniu stosujemy ganciclovir dożylnie przez pierwsze 2-3 tygodnie, następnie w przypadku poprawy stanu ogólnego oraz poprawy wyników badań wirusologicznych, kontynuujemy leczenie doustnie valganciclovirem przynajmniej do łącznie 6 tygodni, z tendencją do wydłużania czasu leczenia nawet do 6 miesięcy. Jednocześnie raz w tygodniu kontrolujemy podstawowe badania laboratoryjne, w tym morfologię krwi z rozmazem białokrwinkowym, enzymy wątrobowe, wskaźniki nerkowe, elektrolity, w razie potrzeby bilirubinę, układ krzepnięcia. Badania wirusologiczne powtarzamy początkowo co tydzień, następnie przy dobrej tolerancji leku i braku powikłań leczniczych co 2-4 tygodni.

Z uwagi na brak dostępności w laboratorium naszego szpitala, badania poziomu gancicloviru we krwi podczas terapii oraz biorąc pod uwagę charakterystykę produktu leczniczego i jego farmakokinetykę [35], celem doboru odpowiedniej, indywidualnej dawki leku dla pacjenta, kontrolujemy systematycznie podczas leczenia wartość kreatyniny lub klirensu kreatyniny w surowicy krwi pacjenta.

### Zalecenia po wypisie z oddziału

#### Badania audiologiczne

Zgodnie z zaleceniami National Deaf Children’s Society u dzieci z wrodzonym zakażeniem CMV należy do ukończenia 3. roku życia oceniać słuch co 3-6 miesięcy, a następnie, aż do ukończenia 7. roku życia, raz na rok.

#### Badanie neurologiczne

U wszystkich dzieci z objawowym zajęciem OUN należy przeprowadzić ocenę neurologiczną jeszcze w trakcie hospitalizacji, najlepiej w 4-6 tyg. życia, a następnie kontynuować ją z częstością uzależnioną od stanu klinicznego pacjenta oraz wyników badań obrazowych OUN. U pozostałych dzieci ocenę neurorozwojową należy przeprowadzić w 6. miesiącu życia, a następnie co najmniej 1 raz na rok w przychodni pediatrycznej.

#### Badanie okulistyczne

Po ustaleniu rozpoznania należy przeprowadzić wyjściowe badanie okulistyczne celem poszukiwania zbliznowaceń siatkówki, będących skutkiem przebytego procesu zapalnego, ale także innych cech zapalenia wirusowego w narządzie wzroku, w tym także aktywnego. Noworodki bez objawów ocznych zakażenia CMV nie wymagają dalszej obserwacji okulistycznej, natomiast dzieci z objawami zakażenia należy raz na rok, aż do ukończenia 5. roku życia, badać pod kątem opóźnionego zapalenia naczyniówki i siatkówki lub jego progresji.

#### Zapobieganie

Brak szczepionki, ograniczona możliwość stosowania terapii przeciwwirusowej u kobiet w ciąży, noworodków i niemowląt sprawia, że istotnym elementem profilaktyki stają się działania zapobiegawcze mające na celu ograniczenie szerzenia się zakażenia HCMV, polegające na szeroko pojętej edukacji zwłaszcza w środowiskach o wysokim narażeniu na to zakażenie. Poniżej przedstawiona tabela (Tabela I) zawiera wskazówki działań profilaktycznych, jednak z uwagi na ryzyko reinfekcji, powinny być one dedykowane nie tylko ciężarnym seronegatywnym, ale także seropozytywnym, zwłaszcza będącym w grupach ryzyka zakażenia nawrotowego, jak opiekunki dziecięce, personel medyczny oddziałów pediatrycznych, matki dzieci w wieku przedszkolnym, a także kobiety w krajach o niskim statusie socjoekonomicznym i słabej edukacji [36, 37, 38]. Mając na uwadze prewencję wrodzonej cytomegalii ważnym jest kontynuowanie badań nad opracowaniem szczepionki przeciwwirusowej oraz mniej toksycznych leków przeciwwirusowych. Ważnym jest także prowadzenie dalszych badań nad stosowaniem HIG i leków przeciwwirusowych u kobiet ciężarnych zakażonych wirusem cytomegalii.

Z uwagi na trudności w interpretacji wyników badań, a także istnienie różnych wariantów postępowania w odniesieniu do diagnostyki, leczenia i profilaktyki, wydaje się zasadnym, by dziećmi z zakażeniem wrodzonym, począwszy od okresu prenatalnego, zajmowali się specjaliści w ośrodkach referencyjnych, posiadający odpowiednie doświadczenie kliniczne i bazę diagnostyczno-leczniczą.

## Podsumowanie

Wirus CMV jest jednym z najbardziej rozpowszechnionych w świecie patogenów wirusowych, jednocześnie stanowi jeden z najczęstszych czynników sprawczych wśród wrodzonych zakażeń wirusowych populacji ludzkiej. Opracowanie skutecznej szczepionki stanowi zatem jeden z priorytetów Światowej Organizacji Zdrowia. Do czasu jej opracowania edukacja jest jedyną formą zapobiegania transmisji CMV. Istnienie udokumentowanej odporności w postaci obecności przeciwciał klasy IgG u ciężarnej nie wyklucza możliwości zakażenia wrodzonego. Przechorowanie zakażenia wirusem CMV nie daje bowiem trwałej odporności pochorobowej, co umożliwia reaktywację infekcji latentnej lub reinfekcję innym serotypem wirusa, z potencjalnym ryzykiem wtórnego zakażenia płodu. Wprowadzenie rutynowych badań przesiewowych u noworodków pozwoliłoby na rozpoznanie większości przypadków dzieci z zakażeniem wrodzonym CMV i zapewnienie im możliwości leczenia oraz dalszego monitorowania. Zapobieganie wrodzonym zakażeniom wirusem CMV jest trudne i mało skuteczne z uwagi na powszechne występowanie wirusa w środowisku człowieka, niską świadomość społeczną tego zagadnienia, szczególnie w zakresie ryzyka zakażenia, możliwych następstw oraz możliwości ograniczenia transmisji.

**Tabela I:** Zalecenia dla kobiet w ciąży (zwłaszcza seronegatywnych − IgG i IgM CMV ujemne), mające na celu zmniejszenie ryzyka zakażeń wrodzonych [31, 32]. Table I. Recomendations for pregnant women (especially seronegative − CMV IgM and IgG negative), in order to decrease the risk of congenital infections [31, 32].

**Należy (*It is recommended to*)**:

Przyjąć, ze wszystkie dzieci poniżej 3 roku życia są potencjalnym źródłem transmisji CMV (*Assume that every child < 3 years is a possible source of CMV trasmision*).

Dokładnie myć ręce mydłem i ciepłą wodą po następujących czynnościach (*Wash your hands carefully with soap and hot water after the following activities*):
− zmiana pieluchy (*Changing a diaper*)
− przebieranie dziecka (*Dressing a child*)
− karmienie (*Feeding a child*)
− kąpiel (*Bathing a child*)
− wycieranie śliny lub wydzieliny z nosa (*Wiping saliva or nasal secr*etion)
− dotykanie zabawek, smoczków, szczoteczki do zębów (*Touching toys, soother, teethbrush*)

Myć i dezynfekować zabawki (*Wash and disinfect toys*)

Przestrzegać ustalonych procedur higienicznych w miejscu pracy (*Follow the hygiene instructions in a place of work*)

**Nie należy (*It is not recommended to*)**:

− Używać tych samych naczyń (*Use the same dishes*)
− Dzielić się jedzeniem (*Share food*)
− Całować dziecko w usta lub w ich pobliżu (*Kiss a child on the lips or near them*)
− Używać wspólnych ręczników (*Use the same towels*)
− Spać w tym samym łóżku (*Sleep in the same bed*)
